# Supplementary material for: Inverse design of 3d molecular structures with conditional generative neural networks
Source: Nat Commun. 2022 Feb 21;13:973. doi: 10.1038/s41467-022-28526-y (PMC8861047; doi:10.1038/s41467-022-28526-y)
Supplement: Supplementary file 1 — Supplementary Information [file 41467_2022_28526_MOESM1_ESM.pdf]

Supplementary Information for

Inverse design of 3d molecular structures with conditional generative neural networks

Niklas W. A. Gebauer,[1,2,3,*] Michael Gastegger,[1,3] Stefaan S. P. Hessmann,[1,2] Klaus-Robert Müller,[1,2] and Kristof T. Schütt[1,2,†]

[1] *Machine Learning Group, Technische Universität Berlin, 10587 Berlin, Germany*
[2] *Berlin Institute for the Foundations of Learning and Data, 10587 Berlin, Germany*
[3] *BASLEARN – TU Berlin/BASF Joint Lab for Machine Learning, Technische Universität Berlin, 10587 Berlin, Germany*

Supplementary Methods

1. 3d grid for molecule generation

We use a grid of candidate positions $\mathbf{G} \subset \mathbb{R}^3$, with a spacing of 0.05 Å. The extent of the grid is limited by a minimum distance d_{min} and a maximum distance d_{max}:

$$\mathbf{G} = \{\mathbf{r} \in \mathbb{R}^3 | \mathbf{r} = (0.05 \cdot x, 0.05 \cdot y, 0.05 \cdot z) \wedge x, y, z \in \mathbb{Z} \wedge d_{\text{min}} \leq ||\mathbf{r}||_2 \leq d_{\text{max}}\}.$$

The limits should be chosen according to the minimum and maximum distances between atoms in the training set that are considered to be neighbors when building atom placement sequences. For our experiments with QM9, we choose $d_{\text{min}} = 0.9$ Å and $d_{\text{max}} = 1.7$ Å.

Furthermore, as in previous work with G-SchNet [1], we utilize a temperature parameter T to control the randomness when sampling from candidate positions:

$$\mathbf{r}'_{\text{next}} \sim \frac{1}{\alpha} \exp\left(\frac{\sum_{j=1}^{i-1} \log \mathbf{p}_j^{\text{next}}}{T}\right) \tag{1}$$

with

$$\mathbf{p}_j^{\text{next}} = p\left(r_{ij} = ||\mathbf{r}_j - \mathbf{r}'_{\text{next}}||_2 | \mathbf{x}_j, \mathbf{y}, Z_i\right), \tag{2}$$

$$\mathbf{r}'_{\text{next}} = \mathbf{r}_{\text{next}} + \mathbf{r}_{\text{focus}}, \tag{3}$$

$$\alpha = \sum_{\mathbf{r}'_{\text{cand}} \in \mathbf{G}} \exp\left(\frac{\sum_{j=1}^{i-1} \log \mathbf{p}_j^{\text{cand}}}{T}\right). \tag{4}$$

Increasing T will increase randomness by smoothing the grid distribution. For sampling, we stick with T=0.1 in this work, which was found to result in accurate yet diverse sets of generated molecules [1].

The very first atom is placed solely based on the predicted distance to the origin token, i.e. the center of mass of the structure about to be generated. Naturally, this distance is not restricted by the same limits as neighboring atoms and thus, for this particular step, we employ a special grid $\mathbf{G}_1 \subset \mathbb{R}^3$ that covers larger distances:

$$\mathbf{G}_1 = \{\mathbf{r} \in \mathbb{R}^3 | \mathbf{r} = (0.05 \cdot x, 0, 0) \wedge x \in \mathbb{N} \wedge ||\mathbf{r}||_2 < 15\}.$$

The maximum distance covered by the grid has been chosen to match with the maximum distance covered in the discretized distance distributions predicted by the model. Due to symmetry, the grid only needs to extend into one direction. Furthermore, the distribution is not smoothed during generation, i.e. we always set T=1.0 when sampling the first atom.

---

* n.gebauer@tu-berlin.de
† kristof.schuett@tu-berlin.de

2. Calculation of relative atomic energy

We define a *relative atomic energy* that describes whether the energy per atom of a 3d conformation is comparatively high or low with respect to other structures in the data set that share the same atomic composition:

$$E^{\mathrm{rel}}(\mathbf{R}_{\leq n}, \mathbf{Z}_{\leq n}) = E(\mathbf{R}_{\leq n}, \mathbf{Z}_{\leq n}) - \hat{E}^{Z}(\mathbf{Z}_{\leq n}). \tag{5}$$

Here, $E(\mathbf{R}_{\leq n}, \mathbf{Z}_{\leq n})$ is the internal energy per atom at zero Kelvin of a molecular structure and $\hat{E}^{Z}(\mathbf{Z}_{\leq n})$ is the expected internal energy per atom of molecules with the same composition in the training data set. A similarly normalized energy has been defined by Zubatyuk et al. [2] for their neural network potential AIMNet. Analogous to their procedure, we predict $\hat{E}^{Z}(\mathbf{Z}_{\leq n})$ from the atomic composition with a linear regression model. The model maps from atomic concentration, i.e. the atomic composition divided by the total number of atoms in the system, to the internal energy per atom at zero Kelvin. In this way, we can compute the relative atomic energy even for structures with compositions that are not included in the training data and treat molecules of different size and composition in a comparable and normalized manner. This allows our model to learn a relation between 3d conformations and their energy that can be transferred across compositions, as can be seen in our experiments where we sample low-energy $C_7O_2H_{10}$ isomers with a model that was trained solely on other compositions (see Figure 4 in the paper).

The internal energy of training structures is provided in QM9 as property "U0". For unrelaxed, generated structures we predict the internal energy with a SchNet model trained on QM9 as explained in the Methods (section IV G). For relaxed, generated molecules we use the internal energy calculated with the ORCA quantum chemistry package [3] (Supplementary Methods S4). Although we relax structures at the same level of theory as the training data , the internal energies obtained with ORCA have a systematic offset compared to the calculations used in QM9. Thus, we estimate this offset and add it to the calculated internal energy. For the relaxed low-energy $C_7O_2H_{10}$ isomers (results in Fig. 4b-d), we re-compute the internal energy of all $C_7O_2H_{10}$ isomers in QM9 with ORCA and take the average difference between the reported internal energies and the re-computed values to estimate the offset (~ -0.0064 eV per atom). For the relaxed low-energy molecules with small HOMO-LUMO gap (results in Fig. 5) we re-compute the internal energy of 1000 randomly sampled structures from QM9 with ORCA and fit a linear regression model to predict the difference between reported internal energies and re-computed values from the atomic composition. This allows to estimate the offset between internal energies from ORCA and the training data for relaxed, generated molecules of arbitrary composition.

3. Calculation of fingerprints

We obtain 1024 bits long binary fingerprints that capture the presence of linear fragments with up to seven atoms with Open Babel [4]. We use version 2.4.1 of Open Babel, where the employed fingerprint is called "FP2" and corresponds to the default choice. Fingerprints are calculated after the SMILES representation of 3d structures are obtained as described in the Methods (section IV F).

4. Relaxation of generated structures with density functional theory

All electronic structure computations were carried out with the ORCA quantum chemistry package [3]. SCF convergence was set to tight and integration grid levels of 4 and 5 were employed during SCF iterations and the final computation of properties, respectively.

Structures were first pre-optimized at the PBE/def2-SVP[5, 6] level of theory and then relaxed at the final B3LYP/6-31G(2df,2p) level [7–10]. We used the same B3LYP parametrization scheme as employed in the Gaussian electronic structure packages. To further accelerate the relaxation procedure, the resolution of identity (RI)[11, 12] and chain of spheres (COS)[13] approximations were used.

The zero point vibrational energies required for the computation of the internal energies were obtained by normal mode analysis performed on the fully relaxed structures using the B3LYP/6-31G(2df,2p) level of theory.

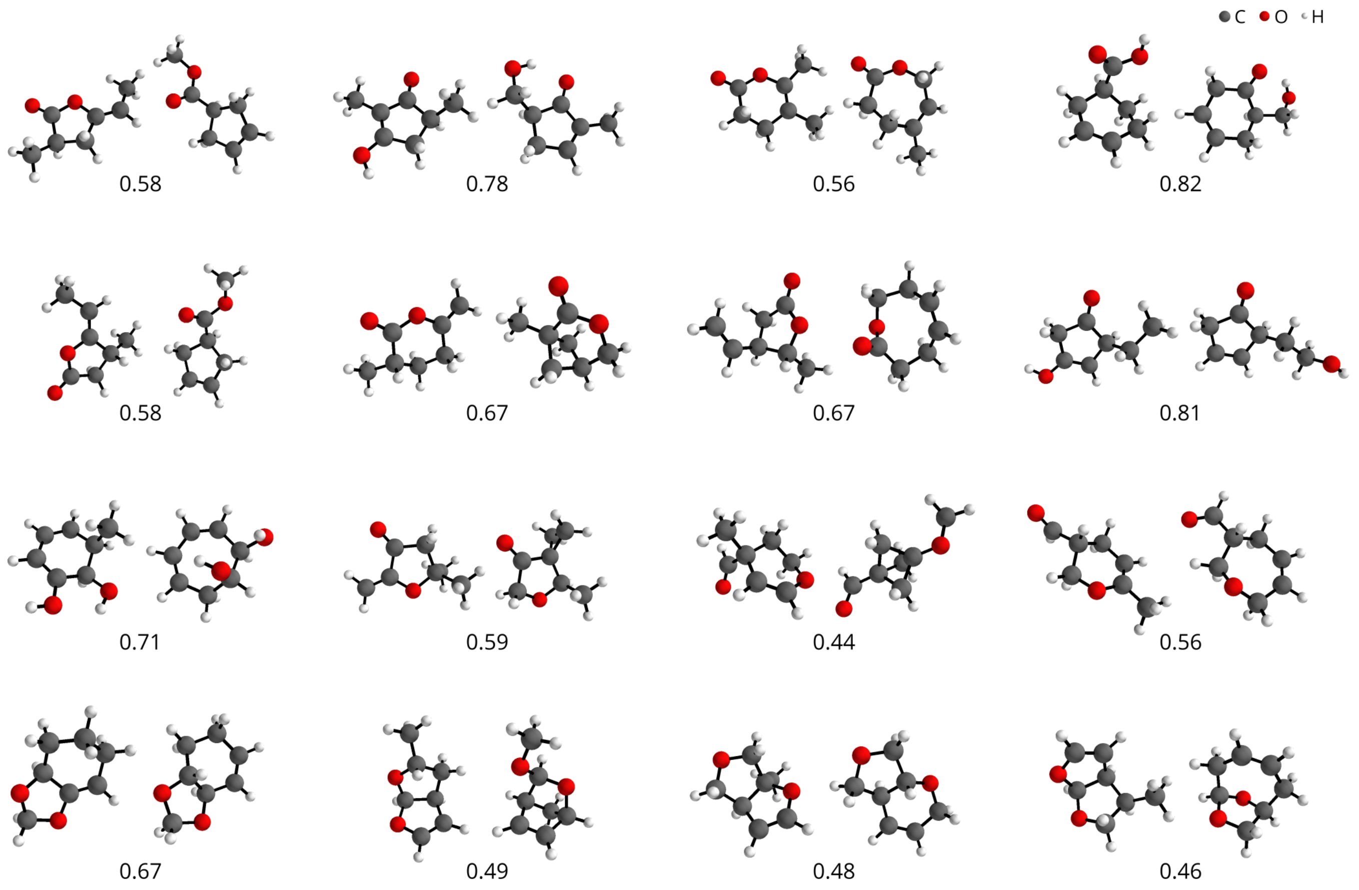

SUPPLEMENTARY FIGURE 1. **Generated novel $C_7O_2H_{10}$ isomers vs. most similar isomers in QM9**. Pairs of generated, novel, low-energy $C_7O_2H_{10}$ isomers (left) and the corresponding most similar $C_7O_2H_{10}$ isomer in QM9 (right) according the Tanimoto similarity of path-based fingerprints (noted below each pair). In the first row, we show pairs corresponding to the novel structures depicted in the first row of Fig. 4d. The remaining structures are uniformly randomly selected from all novel isomers with relative atomic energy $\leq$ -0.05 eV generated by cG-SchNet (target energy -0.1 eV).

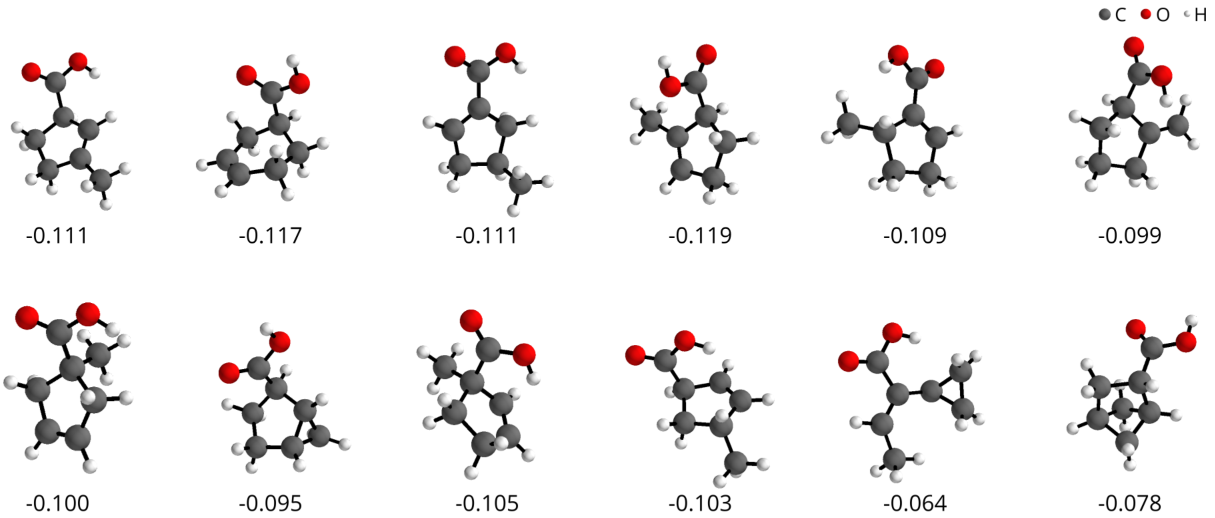

SUPPLEMENTARY FIGURE 2. **Generated novel $C_7O_2H_{10}$ isomers containing carboxylic acid**. The twelve generated $C_7O_2H_{10}$ isomers with relative atomic energy $\leq$ -0.05 eV containing a carboxylic acid group. The molecules were obtained by cG-SchNet with target relative atomic energy -0.1 eV. Relative atomic energies are denoted below each isomer.

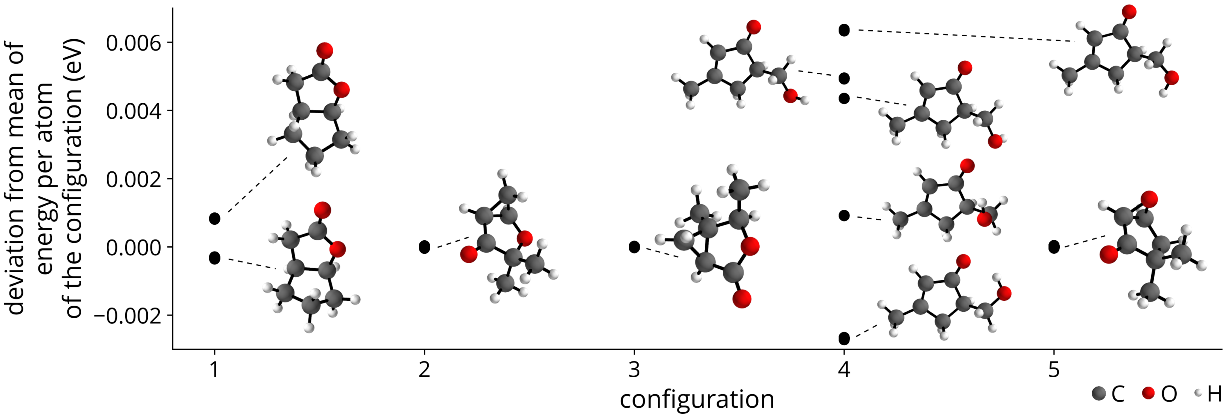

SUPPLEMENTARY FIGURE 3. **Different conformations of the five most generated $C_7O_2H_{10}$ isomers**. For the five most often generated $C_7O_2H_{10}$ isomers (that share the same isomeric SMILES string) from cG-SchNet with target relative atomic energy -0.1 eV we randomly sample 50 of the generated examples. We relax them and report the resulting unique conformations along with their respective deviation from the mean energy per atom of all 50 examples. For three of the five isomers, all examples converge to the same conformation. However, we see that cG-SchNet is capable of sampling multiple conformations whenever there are degrees of freedom, e.g. in isomer number one and isomer number four. Our analysis suggests the path for a possible future adaptation and application of cG-SchNet that is particularly tailored to generative models for 3d molecules, i.e. the targeted generation of conformations for a given (possibly isomeric) graph. To this end, a proper embedding of the molecular graph and several target properties would need to be provided as conditions.

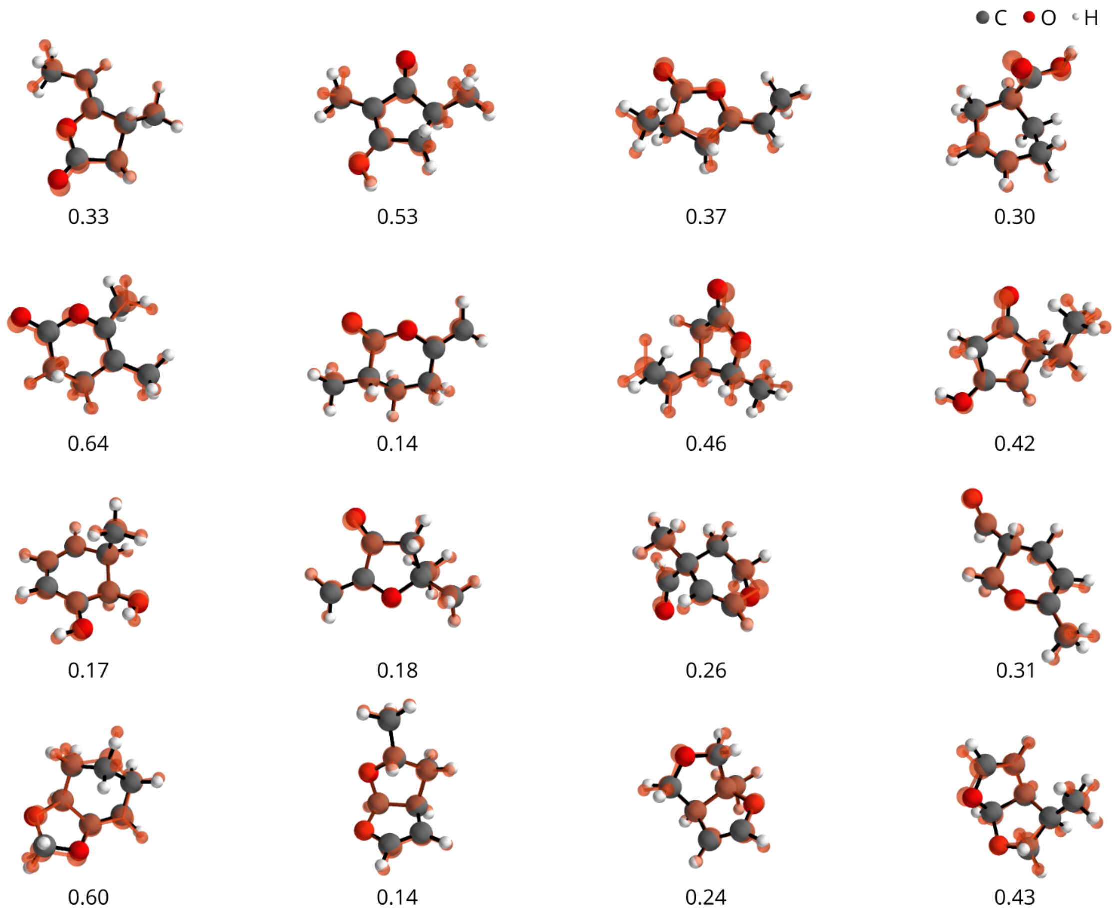

SUPPLEMENTARY FIGURE 4. **Comparison of generated novel $C_7O_2H_{10}$ isomers before and after relaxation**. We show novel, low-energy $C_7O_2H_{10}$ structures as generated by our model and the corresponding closest equilibrium conformation found by relaxation with DFT (orange structures). The root-mean-square deviation between atom positions before and after relaxation is noted below each molecule (in Å). We show the same structures as in Fig. 1.

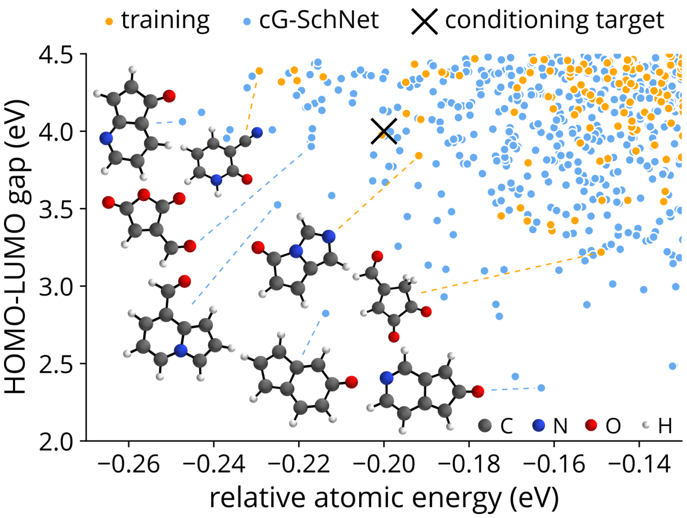

SUPPLEMENTARY FIGURE 5. **HOMO-LUMO gap and relative atomic energy of molecules generated by cG-SchNet and in the training data**. We show the HOMO-LUMO gap and relative atomic energy of molecules close to the conditioning target (black cross) for both the training data set (orange) and the set of unseen and valid structures generated by cG-SchNet (blue). Moreover, three training structures (orange, dotted lines) and five novel, generated molecules (blue, dotted lines) from the borders of the distributions are shown for reference. Here we see how cG-SchNet generalizes to larger structures (i.e. with more than 9 heavy atoms) when sampling molecules with particularly low HOMO-LUMO gap and relative atomic energy.

SUPPLEMENTARY TABLE 1. **Choice of neural network hyper-parameters**. In this work, we trained five cG-SchNet models using different target properties. Each model uses a SchNet network with 128 features, 9 interaction blocks, a cutoff of 10 Å, and 25 centers for the radial basis expansion of distances. For the remaining building blocks, we report the number of neurons per layer in the MLPs and the additional hyper-parameters. Furthermore, we mark which block was used in which model, where model 1 targets isotropic polarizability (results in Fig. 1b), 2 targets molecular fingerprints (Fig. 3a), 3 targets atomic composition and HOMO-LUMO gap (Fig. 3b), 4 targets atomic composition and relative atomic energy (Fig. 4), and 5 targets relative atomic energy and HOMO-LUMO gap (Fig. 5).

Neural network block	Model	Neurons per layer	Additional parameters
isotropic polarizability embedding	1	64, 64, 64	$\lambda_{\min}$: 33.75 a_0^3, $\lambda_{\max}$: 107.95 a_0^3, $\Delta\omega$: 5.3 a_0^3
fingerprint embedding	2	725, 426, 128	—
HOMO-LUMO gap embedding	3, 5	64, 64, 64	$\lambda_{\min}$: 2 eV, $\lambda_{\max}$: 11 eV, $\Delta\omega$: 2.25 eV
relative atomic energy embedding	4, 5	64, 64, 64	$\lambda_{\min}$: -0.2 eV, $\lambda_{\max}$: 0.2 eV, $\Delta\omega$: 0.1 eV
atom count embedding	3, 4	64, 64, 64	$\lambda_{\min}$: 0, $\lambda_{\max}$: 35, $\Delta\omega$: 8.75
composition embedding	3, 4	64, 64, 64	$\mathbf{g}_Z^{\text{comp}}$: 16 features
properties aggregation	all	128, 128, 128, 128, 128	—
type prediction	all	206, 156, 106, 56, 6	—
distance predictions	all	264, 273, 282, 291, 300	L: 300, $\Delta\mu$: 0.05 Å, $\mathbf{g}_Z^{\text{next}}$: 128 features

SUPPLEMENTARY TABLE 2. **Relaxation results**. Results for relaxation of the 100 generated unique unseen molecules closest to the respective target electronic property values. We show the properties on which the respective model was conditioned, the targeted property values, the validity of the relaxed molecules, the median root-mean-square deviation (RMSD) between atom positions before and after relaxation for valid molecules, and the mean absolute error (MAE) between the property values before and after relaxation for valid molecules (i.e. how much the calculated property values of relaxed molecules deviate from the predicted property values of the generated molecules). For the $C_7O_2H_{10}$ isomers sampled with relative atomic energy target -0.1 eV and molecules sampled while targeting HOMO-LUMO gap and relative atomic energy simultaneously, the statistics are calculated from all generated unique unseen molecules instead of the 100 closest (since we relaxed all of them for our analyses in Fig. 4 and Fig. 5).

Conditioning	Target	Validity	RMSD	MAE
isotropic polarizability (a_0^3)	33.75 a_0^3	96%	0.20 Å	2.23 a_0^3
	54.00 a_0^3	99%	0.19 Å	2.35 a_0^3
	74.25 a_0^3	100%	0.23 Å	1.40 a_0^3
	94.50 a_0^3	98%	0.28 Å	0.99 a_0^3
	114.75 a_0^3	100%	0.38 Å	2.95 a_0^3
composition & HOMO-LUMO gap (eV)	$C_7N_1O_1H_{11}$ 5.0 eV	100%	0.32 Å	0.20 eV
	$C_7N_1O_1H_{11}$ 9.0 eV	100%	0.15 Å	0.18 eV
composition & relative atomic energy (eV)	$C_7O_2H_{10}$ -0.1 eV	100%	0.26 Å	0.01 eV
	$C_7O_2H_{10}$ 0.0 eV	100%	0.26 Å	0.01 eV
	$C_7O_2H_{10}$ 0.1 eV	97%	0.17 Å	0.02 eV
HOMO-LUMO gap (eV) & relative atomic energy (eV)	4.0 eV<br>-0.2 eV	100%	0.30 Å	0.33 eV<br>0.03 eV

Supplementary References

[1] N. Gebauer, M. Gastegger, and K. Schütt. Symmetry-adapted generation of 3d point sets for the targeted discovery of molecules. In H. Wallach, H. Larochelle, A. Beygelzimer, F. d'Alché-Buc, E. Fox, and R. Garnett, editors, *Advances in Neural Information Processing Systems 32*, pages 7566–7578. Curran Associates, Inc., 2019.

[2] R. Zubatyuk, J. S. Smith, J. Leszczynski, and O. Isayev. Accurate and transferable multitask prediction of chemical properties with an atoms-in-molecules neural network. *Sci. Adv.*, 5(8):eaav6490, 2019.

[3] F. Neese. The ORCA program system. *WIREs Comput. Mol. Sci.*, 2(1):73–78, 2012.

[4] N. M. O'Boyle, M. Banck, C. A. James, C. Morley, T. Vandermeersch, and G. R. Hutchison. Open babel: An open chemical toolbox. *J. Cheminf.*, 3(1):33, Oct 2011.

[5] J. P. Perdew, K. Burke, and M. Ernzerhof. Generalized gradient approximation made simple. *Phys. Rev. Lett.*, 77: 3865–3868, 1996.

[6] F. Weigend and R. Ahlrichs. Balanced basis sets of split valence, triple zeta valence and quadruple zeta valence quality for H to Rn: Design and assessment of accuracy. *Phys. Chem. Chem. Phys.*, 7(18):3297–3305, 2005.

[7] A. D. Becke. Density-functional thermochemistry. III. the role of exact exchange. *J. Chem. Phys.*, 98:5648–5652, 1993.

[8] C. Lee, W. Yang, and R. G. Parr. LYP correlation: Development of the Colle-Salvetti correlation-energy formula into a functional of the electron density. *Phys. Rev. B*, 37(2):785, 1988.

[9] S. H. Vosko, L. Wilk, and M. Nusair. Accurate spin-dependent electron liquid correlation energies for local spin density calculations: a critical analysis. *Can. J. Phys.*, 58(8):1200–1211, 1980.

[10] P. J. Stephens, F. J. Devlin, C. F. Chabalowski, and M. J. Frisch. Ab initio calculation of vibrational absorption and circular dichroism spectra using density functional force fields. *J. Phys. Chem.*, 98(45):11623–11627, 1994.

[11] K. Eichkorn, O. Treutler, H. Öhm, M. Häser, and R. Ahlrichs. Auxiliary basis sets to approximate Coulomb potentials. *Chem. Phys. Lett.*, 240(4):283–290, 1995.

[12] O. Vahtras, J. Almlöf, and M. W. Feyereisen. Integral approximations for LCAO-SCF calculations. *Chem. Phys. Lett.*, 213(5-6):514–518, 1993.

[13] F. Neese, F. Wennmohs, A. Hansen, and U. Becker. Efficient, approximate and parallel hartree–fock and hybrid dft calculations. a 'chain-of-spheres' algorithm for the hartree–fock exchange. *Chem. Phys.*, 356(1-3):98–109, 2009.
